# Domestication of newly evolved hexaploid wheat—A journey of wild grass to cultivated wheat

**DOI:** 10.3389/fgene.2022.1022931

**Published:** 2022-10-03

**Authors:** Sasha Gohar, Muhammad Sajjad, Sana Zulfiqar, Jiajun Liu, Jiajie Wu, Mehboob-ur- Rahman

**Affiliations:** ^1^ Plant Genomics and Molecular Breeding Laboratory, National Institute for Biotechnology and Genetic Engineering, Faisalabad, Pakistan; ^2^ Department of Biotechnology, Pakistan Institute of Engineering and Applied Sciences (PIEAS), Islamabad, Pakistan; ^3^ Department of Biosciences, COMSATS University Islamabad, Islamabad, Pakistan; ^4^ State Key Laboratory of Crop Biology, Shandong Agricultural University, Tai'an, Shandong, China

**Keywords:** domestication, hybrid wheat, NGS, CRISPR, genomic selection (GS), climate change, food security

## Abstract

Domestication of wheat started with the dawn of human civilization. Since then, improvement in various traits including resistance to diseases, insect pests, saline and drought stresses, grain yield, and quality were improved through selections by early farmers and then planned hybridization after the discovery of Mendel’s laws. In the 1950s, genetic variability was created using mutagens followed by the selection of superior mutants. Over the last 3 decades, research was focused on developing superior hybrids, initiating marker-assisted selection and targeted breeding, and developing genetically modified wheat to improve the grain yield, tolerance to drought, salinity, terminal heat and herbicide, and nutritive quality. Acceptability of genetically modified wheat by the end-user remained a major hurdle in releasing into the environment. Since the beginning of the 21^st^ century, changing environmental conditions proved detrimental to achieving sustainability in wheat production particularly in developing countries. It is suggested that high-tech phenotyping assays and genomic procedures together with speed breeding procedures will be instrumental in achieving food security beyond 2050.

## Introduction

Wheat (*Triticum aestivum* L.) is cultivated in 89 countries to feed around 2.5 billion people—one-fifth of the total world population. Bread wheat is grown on about 95% of the total wheat cropped area while the remaining 5% area is covered by the durum wheat (Mastrangelo and Cattivelli, 2021). The contribution of durum wheat in total wheat production is also around 5%.

Wheat domestication and human civilization evolved simultaneously in the history of mankind. Among the cereals, it is one of the most important crops with relatively more potential to get adapted in challenging environments. With the increasing food demand and depleting agricultural land, it is pivotal to enhance the grain yield in a sustainable way to feed the increasing human population beyond 2025. Nearly 100% increase in wheat production is inevitable to meet the global food requirements by the end of 2050 (Senker, 2011). Concerning the advancements in research and development, all the major events and technologies that paced up wheat research and have a plethora of contributions towards wheat improvement after its domestication as a cereal crop have been described in this article.

## Origin and domestication of wheat

**FIGURE 1:**
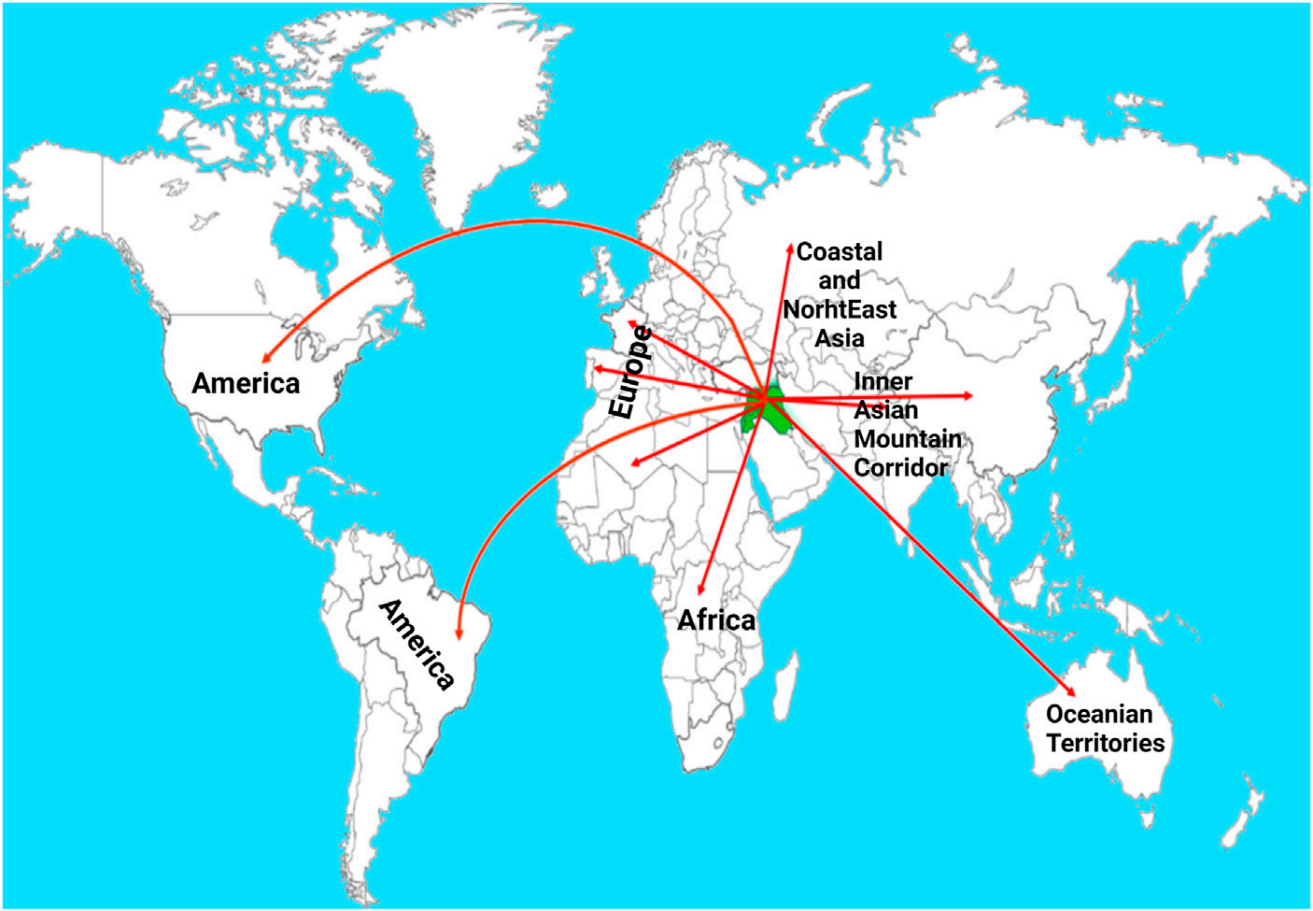
Routes showing the migration of wheat from the center of origin-Fertile Crescent-to other continents of the world. The green color indicates Fertile Crescent and the red lines indicate different known routes (Pont et al., 2019). Post-Domestication to Mendel’s Era.

The hexaploid wheat (AABBDD) contains three different genomes each derived from different diploid species *viz.*, *Triticum urartu* (AA genome), *Aegilops speltoides* (controversial, BB genome), and *Aegilops tauschii* (DD genome) (Feldman et al., 1995; Nesbitt and Samuel, 1996). According to the archeological records, wheat originated in Southeast Turkey. Initially, the progenitor species containing AA and BB subgenomes were discovered (Aaronsohn, 1910) and these were hybridized followed by a doubling of chromosomes which resulted in tetraploid fertile wheat, *T. turgidum* (AABB) (v. Tschermak and Von, 1914). Then the *T. turgidum*, wild emmer, was domesticated in Fertile Crescent. Afterward, *T. turgidum* hybridized with a diploid specie A. *tauschii* (Kihara, 1944; McFadden, 1944; McFadden and Sears, 1946) which resulted in the formation of hexaploid wheat (AABBDD). The hexaploid bread wheat evolved in the Fertile Crescent (Figure 1)*.* It is worth mentioning that tetraploid ancestors spread into the natural range of diploid species *Ae. tauschii.* Because of its high acceptance as an ultimate source of calories, it was spread into different parts of the world *via* different routes (Figure 1). After domestication, hexaploid wheat was cultivated and selected in diverse geographical regions for centuries which resulted in present-day cultivated bread wheat (McFadden and Sears, 1946; Dubcovsky and Dvorak, 2007). Among diploids, einkorn wheat, *Triticum monococcum*, is considered the first domesticated hulled wheat. The historical record shows that it was domesticated  12,000—c. 8,500 years ago in the Pre-Pottery Neolithic period (Zaharieva and Monneveux, 2014). However, cultivated tetraploids *Triticum dicoccum* (wheat emmer) and *Triticum durum* (tetraploid durum), both arose from wild ancestors.

Initially, the wheat was spread to Greece, Cyprus, India, and Egypt followed by other countries around the world (Cooper, 2015). Domesticated wheat had large-sized hulled seeds attached to the ear as compared to its wild species. The wheat grain in primitively cultivated species was long, thin, and small in size. The first naturally mutated traits in the wild germplasm were non-brittle rachis and naked grain that were responsible for the domestication of wheat (Pourkheirandish et al., 2018). The process of natural selection played a significant role in shaping the present-day cultivated wheat. Selections were made by the early farmers on the basis of phenotypic traits such as grain size, grain color and non-shattering type (Eckardt, 2010). Seeds from the best plants were used to grow the next generation while inferior quality seeds were discarded (Charmet, 2011).

During the 1800s, several wheat varieties were developed by selecting superior lines by the wheat breeders. Wheat growers or distributors were used to select different wheat spikes with desirable quality followed by assigning a new fancy name to the selected spikes such as ‘Thomas Rust Resistant’ for wheat variety resistant to rust and ‘Landretti’s Hard Winter’ for resistance to changing climate (Spennemann, 2001).

In the 18^th^ century, wheat ‘rust’ was scientifically described for the first time (Eriksson and Henning, 1896). Efforts were made to improve wheat varieties mainly for high yield and resistance to diseases. Similarly, wheat breeding for protein content and baking quality was initiated by William James Farrer (Wrigley et al., 1981). Then in 1873, interspecific hybridization between rye and wheat was successfully made, and thereafter enormous valuable hybrids of wheat showed significant improvements in yield and other traits such as early maturity, rust-resistance with stiff straws, gluten content, and non-shattering traits were developed (Beach, 1923).

## Wheat genetics in post-mendelian era

In the late 19^th^ century, Mendel published data pertaining to his historic experiments conducted on pea plants. In the early 1900s, his work was re-discovered which provided him recognition as the “Founder of Genetics” (Wrigley et al., 1981)**.** Since then, breeding based upon scientific knowledge started and the whole research perspective was shifted towards improving desirable traits such as plant height, seed color, and seed shape instead of the plant as a whole (Biffen, 1905). In 1916, the first hard wheat variety ‘Yeoman’ having low protein content was developed through hybridization. It was discovered that for achieving genetic stability, more than 10 generations were required for fixing the traits (Bajaj, 1990). In 1920, the stem rust gene, *Sr2* was incorporated into wheat from tetraploid emmer wheat (Singh and McIntosh, 1984). Following several hybridization experiments, Italian landraces and inbred lines were crossed with the Japanese variety ‘Akakomugi’. Resultantly, new varieties harboring improved resistance to rust diseases, early flowering, and early maturing were developed. These varieties were used in other wheat breeding programs, and laid down a firm foundation for achieving green revolution (Salvi et al., 2013). During the early period of hybridization-based breeding programs, varieties depicting high yield potential were developed without focusing on improving resistance to biotic stresses. Another winter wheat variety ‘Turkey red’ was also developed and cultivated in the United States (Olmstead and Rhode, 2002). Drought resistance was incorporated from landraces to ‘Aragon 03’ (Royo and Briceño-Félix, 2011). Later on, wheat varieties with high Zn and protein content as well as biotic and abiotic stress tolerance were also developed through conventional breeding.

## The era of mutagenesis

By the process of mutagenesis, novel genetic variability in plants was induced by exposing them to physical or chemical mutagens (de Oliveira Camargo et al., 2000). Over the last century, physical mutagens, for example, gamma rays, UV rays, fast neutrons, and the chemical mutagens such as sodium azide, N-methyl-N-nitrosourea, ethyl methansulfonate and hydrogen fluoride have been widely used. Biological mutagens like Agrobacterium are also being used (Krishnan et al., 2009). Recently, the mutant population was developed by exposing the seeds of a wheat cultivar ‘Punjab-11’ to gamma-rays. The developed mutants were found to be resistant to either leaf rust, yellow rust or, stem rust (Hussain et al., 2021). Few of these mutants also demonstrated high grain quality traits as compared to wild type (Zulfiqar et al., 2021).

**FIGURE 2:**
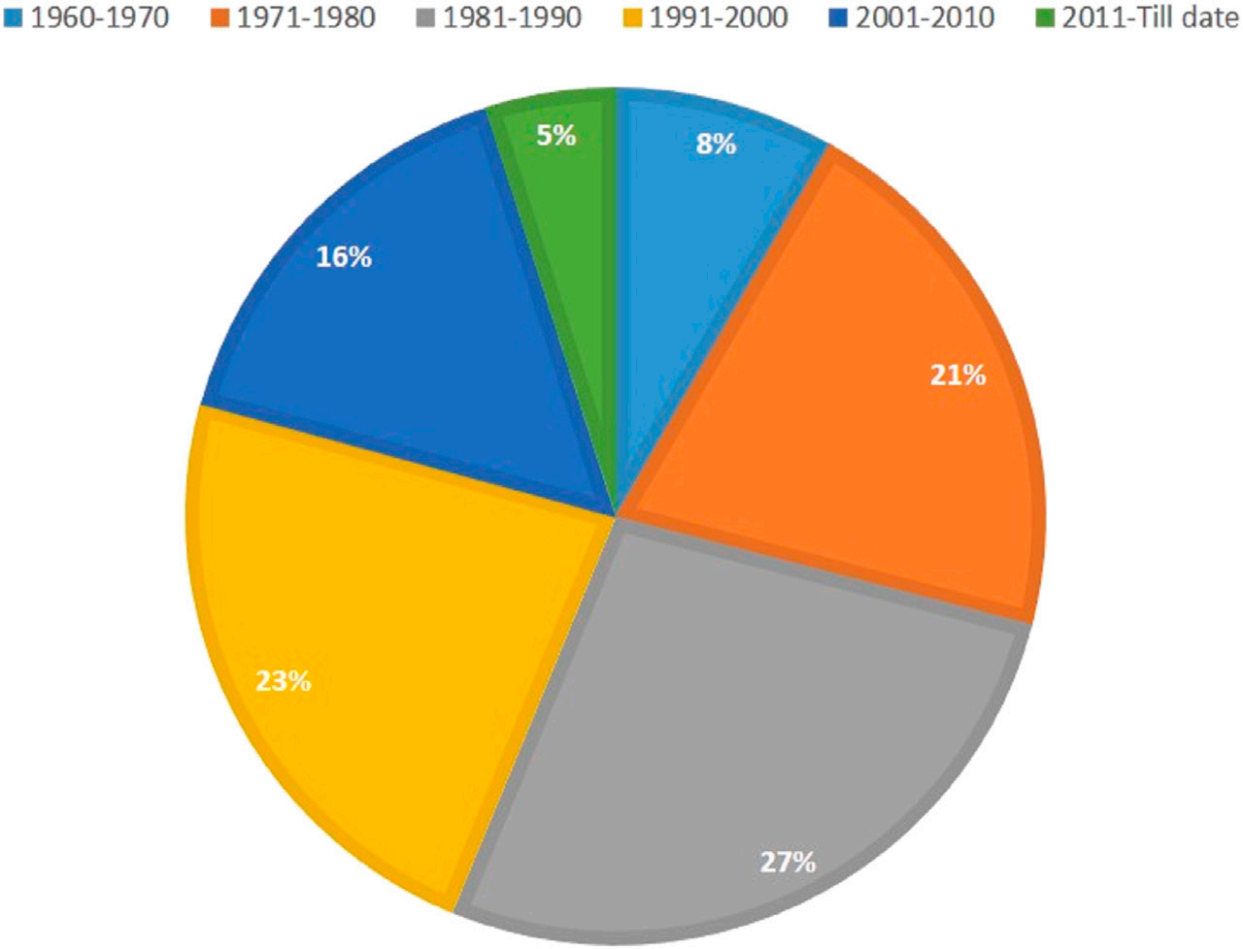
Percentage of mutant wheat varieties developed through mutagenesis in various decades (Data source: https://nucleus.iaea.org/sites/mvd/SitePages/Search.aspx).

Mutation breeding techniques were resurrected during early years of the 21^st^ century due to a better understanding of mutagens, their use, the process of mutagenesis, and its application in related disciplines. Nowadays, traditional approaches being used for the selection of mutants in second and third generations have provided high yielding as well as better quality varieties (Singh and Balyan, 2009; Albokari, 2014). Consequently, a huge number of varieties with improved traits have been released through mutation induction which reveals the economic impact of this technology (Micke et al., 1990; Jankowicz-Cieslak et al., 2017). To date, ∼ 3400 mutant varieties have been produced through mutagenesis directly or indirectly, including 265 varieties of bread wheat (https://nucleus.iaea.org/sites/mvd/SitePages/Search.aspx) (Figure 2). However, the majority of the varieties (∼85%) are the result of mutation inductions through gamma rays. All these varieties released through mutation breeding are high yielding, with better tolerance to pests, diseases, and biotic and abiotic stresses.

The use of chemical mutagens on a large scale has been started in 2000. Ethyl methanesulphonate (EMS) –the most commonly used mutagen-generates random point mutations in the entire genome (Brini and Masmoudi, 2014). For example, a single nucleotide polymorphism (SNP) in *Lr21* was identified in one of the resistant mutants (to leaf rust) of ‘NN-Gandum-1’. This mutation caused a substitution of glutamic acid with alanine (Hussain et al., 2018). Few of these mutants also demonstrated better tolerance to drought stress as compared to wild type (Zahra et al., 2021). It is suggested that mutagenesis experiments are effective in inducing useful mutations in wheat which can be used in forward and reverse genetic studies for gaining insights into the important biological traits of the complex wheat genome.

In the beginning of the 21^st^ century, an advanced method, Targeting Induced Local Lesions IN Genomes (TILLING) was used for identifying point mutations in targeted genes of mutants and or genetic stocks (Henikoff and Comai, 2003). In 2009, through TILLING, complete waxy wheat was bred by crossing *Wx-A1* and *Wx-D1* truncation mutant whereas *Wx-B1*was naturally null in both of them (Dong et al., 2009). Similarly, a waxy gene *GBSS-I* (granule-bound starch synthase I) in near null waxy mutant was identified (Slade et al., 2012). In 2012, the wheat mutant gene *MNR220* was identified that carried resistance locus to powdery mildew and other types of rust. The mutant population resulted after TILLING with many novel alleles that could be a good genetic resource for improvement of wheat (Chen et al., 2012). Novel genetic variations in the *SBEIIa gene* for amylose content*,* *TaAGP* gene for starch biosynthesis and, *TaMlo* gene for durable resistance against powdery mildew were also identified through TILLING (Slade et al., 2012; Acevedo-Garcia et al., 2017; Guo et al., 2017).

## Introgression wheat breeding

Among cereals, a lot of introgression work was done on wheat (Dempewolf et al., 2017), resulting in significant improvement in the genetic diversity of wheat (Lu and Ellstrand, 2014). The genetic sources of wheat, consisting of wild relatives, landraces, and close relatives have contributed significantly to adding novel genetic variations to modern wheat cultivars (Molnar-Lang et al., 2016). In 1930, stem rust resistance *Sr2* gene was introgressed into cultivated wheat from its wild relative emmer wheat cultivar “Yaroslav” (McFadden and Sears, 1946). Several genes against biotic stresses such as Ug99 were found in Aegilops. Genes for stem rust including *Sr33, Sr45, Sr45, Sr46,* and *SrTA1662* were introgressed and localized into the genome of cultivated wheat (Olson et al., 2013).

*Secale cereale*, commonly known as rye, is one of the most important wheat relatives which was used for incorporating several genes into the cultivated wheat. After hybridization with rye, translocations and substitutions played a role in transferring genes responsible for high yield and disease resistance (Rabinovich, 1998). The most important *non-Triticum* introgressions in the wheat genome were 1BL/1RS, 1DL/1RS and 1AL/1RS translocations that contained biotic and abiotic stress resistance genes (Rabinovich, 1998; Mago et al., 2015). The 1BL/1RS translocation between wheat chromosome ‘1B’ and rye chromosome ‘1R’ carrying genes for leaf rust (*Lr26*), stem rust (*Sr31*), stripe rust (*Yr9*), and powdery mildew (*Pm8*) improved the resistance to fungal diseases in wheat (Singh et al., 1990; Friebe et al., 1996; Friebe et al., 1999; Baffes, 2005). The wheat lines containing the 1RS chromosomal arm exhibited a substantial increase in the root length and spike length (Liu et al., 2020a). Likewise, introgression of 4R and 6R chromosomes from rye cultivar ‘Kriszta’ resulted in a significant increase in protein content (Schneider et al., 2016). A novel stem rust resistant gene *Sr59,* yellow rust resistant gene *Yr83,* and powdery mildew resistant gene *Pm56* from rye were introgressed into wheat as a 2DS:2RL and 6AL:6RS Robertsonian translocations, respectively (Table 1) (Rahmatov et al., 2016; Hao et al., 2018)

**TABLE 1:** List of genes/translocations successfully transferred from wild relatives into wheat.

Sr #	Donor species	Name of gene	Translocation chromosome	Reference
1	*Aegilops speltoide*	Stem rust/*Sr39*	2S/2B	Niu et al. (2011)
2	*Aegilops speltoide*	Tan spot *TsrAes1* Septoria nodorum blotch *SnbAes1*	2S/2B	Zhang et al. (2019)
3	*Aegilops sharonensis*	*Lr56/Yr38*	T6AS.6AL-6S^sh^/6L	Marais et al. (2010)
4	*Thinopyrum intermedium*	Wheat streak mosaic virus *Wsm3*	T7BS·7S#3L	Danilova et al. (2017)
5	*Dasypyrum villosum*	Stem rust *Sr52*	T6AS·6V#3L	Li et al. (2019)
6	*Hordeum vulgare*	β-glucan synthesis *HvCslF6*	T7AS·7HL, T7BS·7HL and T7DS·7HL	Danilova et al. (2019)
7	*Elymus tsukushiensis*	*Fusarium head blight Fhb6*	1E^ts^#1S	Cainong et al. (2015)
8	*Hordeum vulgare*	Salt tolerance β-D-glucan	7BS.7H	Türkösi et al. (2018)
9.10	*Hordeum vulgare*	cellulose synthase-like F6 gene (*CslF6*)	A complete set of six compensating RobT chromosomes	Danilova et al. (2018)
10.11	*Secale cereal*	Powdery mildew resistance gene *Pm56*	6AL.6RS	Hao et al. (2018)
11.12	*Secale cereal*	*Stem rust Sr59*	2DS**.**2RL	Rahmatov et al. (2016)
12.13	*Dasypyrum villosum*	Glume ridges (*Bgr*-*V1*) photoperiod response gene (*Ppd*-*V1*)	2VS.2DL	Rahmatov et al. (2016)
13.14	*Secale cereal*	stripe rust and powdery mildew	1BL.1RS	Ren et al. (2018)
14.15	*Secale cereal*	stripe rust and powdery mildew	1RS.1BL	Ren et al. (2017)
15.16	*Secale cereal*	Greenbug resistance genes *Gb2* and *Gb6*	1AL.1RS	Lu et al. (2010)
16.17	*Secale cereal*	Drought-responsive genes	1BL.1RS	Jang et al. (2017)
17.18	*Secale cereal*	Stripe Rust Yr24/26	1RS.1BL	Yang et al. (2016)
18	*Th. bessarabicum*	High Fe and Zn contents	6EbS·6DL	Ardalani et al. (2016)
19	*Th. Elongatum*	Flour Quality genes	1AS.1EL	Tanaka et al. (2017)
20	*Aegilops searsii*	Powdery mildew *Pm57*	2Ss#1/2B	Liu et al. (2017)
21	*Aegilops speltoides*	Stem rust *Sr39*	2S/2B	Zhang et al. (2019)
22	*Th. elongatum*	Wheat streak mosaic virus *Wsm3*	2E/2B	Zhang et al. (2018b)
23	*T. durum*	*Yr7*	2BL	Marchal et al. (2018)
24	*T.spelta*	*Yr5*	2BL	Marchal et al. (2018)
25	*P. Huashania*	All disease resistance and agronomic traits	2Ns/2D	Bai et al. (2020)
26	*Secale cereal*	Aphid and Hessian fly	3DL.3RS and 5AL.5RS	Johansson et al. (2020)
27	*Thinopyrum elongatum*	*Fusarium head blight*	7E.7D	Fedak et al. (2021)
28	*Secale cereal*	Pest and disease resistance	1BS	Przewieslik-Allen et al. (2019)
29	*Triticum timopheevii*	Agronomic traits	2At.A5, 6At.A9, 7G.B4	King et al. (2022)
30	*Aegilops tauschii*	Quality traits, Resistance to biotic and abiotic stresses	5D.5B	Othmeni et al. (2022)

Deficiency of essential micronutrients also called as hidden hunger has affected around two billion people in the world. As a major staple crop, wheat provides almost 20% protein and energy to mankind. Hence, wheat is an ideal candidate for biofortification. Improvement of protein content in wheat grain has been remained a major breeding objective of several wheat groups around the globe. In wild emmer wheat, a *Gpc-B1* locus was discovered which can enhance the content of protein, Zn and Fe; hence can be incorporated into cultivated wheat to increase its nutritional value (Uauy et al., 2006). This gene was introgressed into two cultivars HUW468 and HUW234 through marker assisted backcrossing (Vishwakarma et al., 2014; Mishra et al., 2015). The introgression of *Gpc-B1* gene for increasing grain protein content has also been achieved in 10 elite wheat cultivars (Kumar et al., 2011). Moreover, introgression of *Gpc*-*B1* gene was also performed in different spring wheat cultivars (Carter et al., 2012; Tabbita et al., 2012; Eagles et al., 2014), where grain protein contents were substantially increased. Tolerance to salt, and (1,3;1,4))-β-D-glucan content were also transferred in wheat as a 7BS.7HS wheat/barley Robertsonian translocation (Türkösi et al., 2018). Recombinant inbred wheat lines were developed by introgression with *A. caudate;* newly developed lines showed improved disease resistance. Also, resistance to the take-all disease was incorporated in the 2NS/2D substitution line of bread wheat by making a cross with *Psathyrostachys huashania* Keng (Table 1) (Bai et al., 2020). Recent technological advancements in genomics and cytogenetics offer new avenues for transferring alien genes to wheat, avoiding issues like linkage drag.

## The era of the green revolution in wheat production

In 1950, photoperiod insensitive (*ppd1* and *ppd2*) genes were transferred into wheat for expanding germplasm usage globally (Rajaram, 2001). After the wheat rust epidemic (1951–1954) in North America, research for the development of rust-resistant modern cultivars was initiated by the CIMMYT (International Maize and Wheat Improvement Center, Mexico). Another initiative that prevented the outbreak of the famine in 1970s was the introduction of genes which resulted in a significant reduction of plant height in wheat. These genes were derived from a wheat genotype ‘Norin-10’. The semi-dwarf “Norin-10” was about 60 cm in height, more responsive to nitrogen fertilizer, resistant to rust, and had lodging resistance. Norin-10 was estimated to be cultivated on 15–18 million acres worldwide (Reitz and Salmon, 1968). The genes responsible for conferring short height were named *Rht-B1* and *Rht-D1* (Khush, 1999). The introduction of these genes in wheat paved the way for wheat breeding aimed at enhancing yield potential which ultimately helped in alleviating hunger and poverty across the globe. These varieties brought a green revolution in several developing countries like Pakistan, India, Turkey, Afghanistan, etc. Dr. Norman Borlaug was awarded Nobel Peace Prize for his brilliant work (Borlaug, 2007).

**FIGURE 3:**
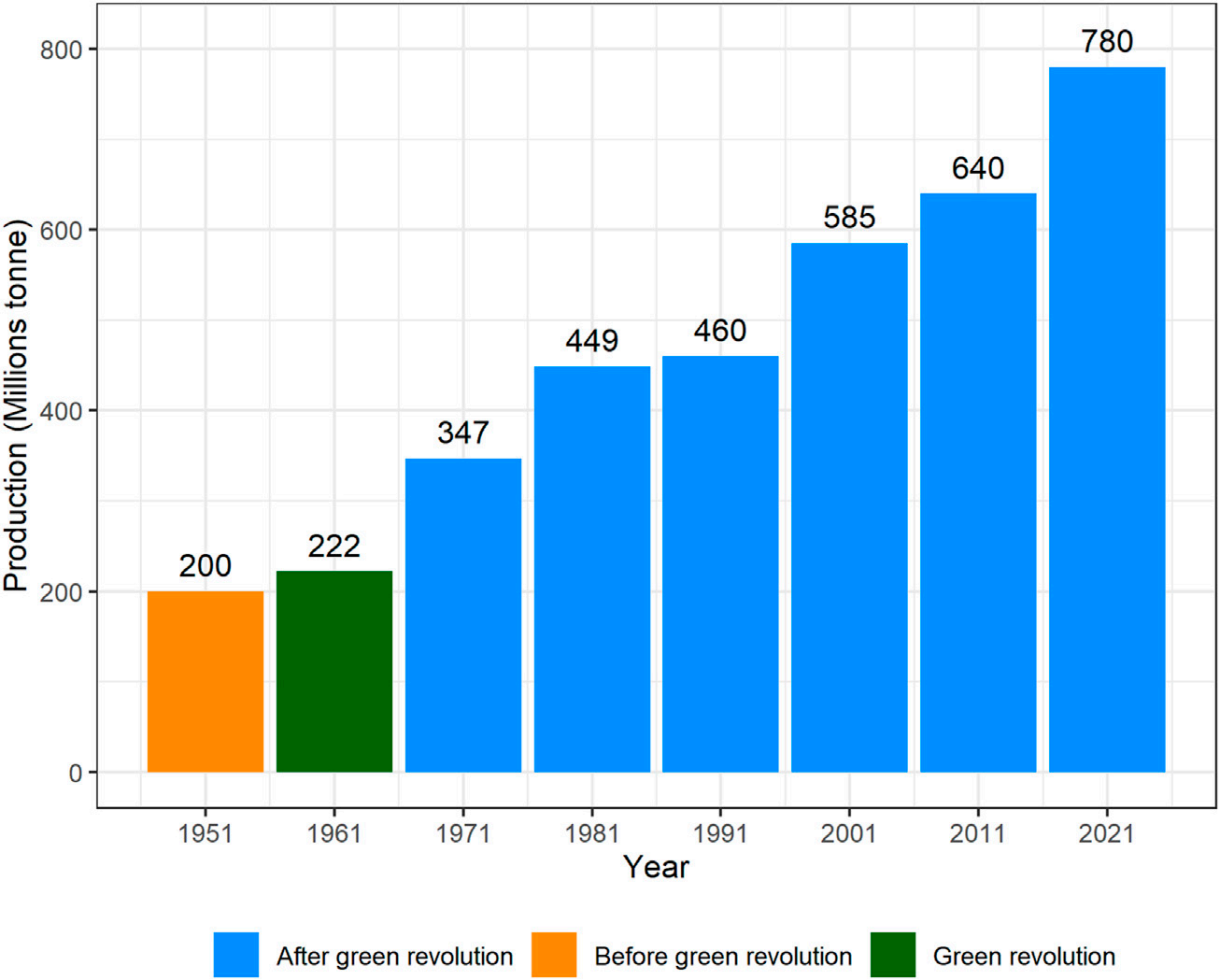
Worldwide wheat production after the green revolution (FAOSTAT, 2022).

**FIGURE 4:**
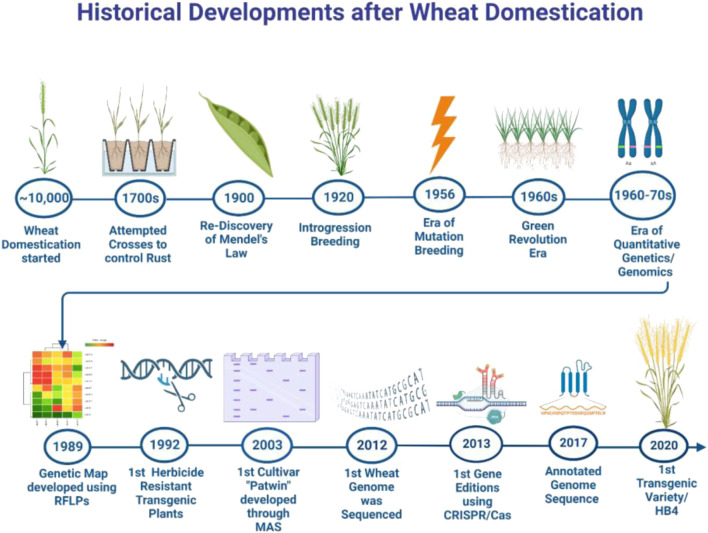
Timeline of historical developments in wheat research since its domestication.

After the Green revolution, the wheat yield increased many folds (Figure 3), however, the nutritional quality was compromised (Ortiz-Monasterio et al., 2007)**.** Increasing the nutritional quality of cultivated wheat became another challenge for wheat breeders. Also, the other disadvantage of the green revolution was that many old varieties disappeared, and many of these led to extinction (Eliazer Nelson et al., 2019). Almost 63% of wheat varieties released in the 21^st^ century contain “Green revolution” alleles (Würschum et al., 2017). But the performance of varieties having these alleles was not satisfactory in dry and warm regions owing to arrested growth of coleoptile and seedling emergence (Rebetzke et al., 2014). An alternative dwarfing allele, *Rht18* was identified that has no impact on coleoptile length, and hence can be used to replace previous dwarfing genes in target environments (Pearce, 2021). In the post-green evolution era, stem rust had also threatened these short-statured varieties. So, during this period, *Sr2, Sr5*, *Sr6*, *Sr7a*, *Sr7b*, *Sr8a*, *Sr9b*, *Sr9d*, *Sr9e*, *Sr9g*, *Sr10*, *Sr11*, *Sr12*, *Sr17*, *Sr24*, *Sr26*, *Sr30*, *Sr31* and *Sr36* genes were incorporated into wheat (Knott, 1988). However, later on, new devastating rust races evolved that became a serious threat to wheat germplasm, globally. Stem rust race Ug99 and its variants caused 80–100% yield losses in different countries of the world (Khan et al., 2013). These fast-evolving races of rust put the attention of breeders toward pyramiding two or three major genes to induce durable resistance in wheat. Later on, different varieties having minor genes for stem rust, leaf rust, and yellow rust were developed to avoid the issue of resistance breakdown (Figure 4).

## Hybrid wheat: Future prospects

Breeding hybrids remains instrumental in uplifting the production of several crop species including corn. There is a dire need to breed for adding resilience to changing environments and high-yielding wheat varieties to address the pressing issue of global food security. The exploitation of hybrid vigor may entail the desired increase in wheat yield (Longin et al., 2013), biotic/abiotic stress tolerance as well as grain quality (Gowda et al., 2012; Longin et al., 2013; Longin et al., 2014; Mühleisen et al., 2014; Longin et al., 2015; Jiang et al., 2017; Thorwarth et al., 2018);

The development of wheat hybrid is entirely dependent upon manual crossing between the two inbred lines. Efforts to develop hybrid wheat have a long history, however, the success rate is very slow because of its autogamous nature and tedious procedure of making crosses. Heterosis was first time reported in wheat in 1919, since then several efforts have been attempted to establish a well-defined hybrid production system in wheat (Freeman, 1919; Pickett, 1993). During the 1950s and 1980s, hybrid wheat breeding showed 10% mid-parent heterosis for grain yield. For hybrid development, cytoplasmic male sterility (CMS) and chemical hybridizing agents (CHA) were two methods practiced for removing pollens of the lines used as female (Kihara, 1951). The CMS and restorer genes were derived from *T. timopheevii* (Wilson, 1962). The research efforts being done in hybrid breeding could not be continued in real spirit owing to the maintenance of three lines, and some genetic issues related to complete restoration of fertility as well as the undesirable effect of alien cytoplasm (Singh et al., 2010).

Recent years witnessed the cloning of several male sterility genes which provided new tools for producing hybrid seeds in wheat. The dominant male-sterility gene *Ms2* present in common bread wheat facilitated the evolution of several lines and cultivars, and could further be utilized in developing a high-throughput hybrid production system (Ni et al., 2017). The cloning of a nuclear recessive male sterility gene *Ms1* also provides a new resource for large-scale commercial hybrid seed production (Wang et al., 2017). In addition, orthologous male sterile genes identified in grasses could help to understand the male sterile trait in wheat. In total, three homoeoalleles of *OsNP1* showed similar expression to *OsNP1* and *ZmIPE1* in wheat. The optimized CRISPR/Cas9 mediated triple homozygous *Tanp1* mutant displayed complete male sterility and only one wild type of *TaNP1* gene was sufficient for the maintenance of male fertility (Li et al., 2022). This work provided an optimized CRISPR/Cas9 vector system in wheat, elucidated the highly conserved function of *TaNP1* genes, and produced complete male sterile mutants which can be used in hybrid seed production. This genetic male sterile line will lead to two-line hybrid seed production in wheat. The two-line system will be free of negative effects associated with alloplasms, cytoplasm, and restorer line. This two-line system is relatively simple and potentially more efficient as compared to the existing 3-lines hybrid system; which demands a high cost of maintenance with a limited level of success in restoring fertility in F1 hybrids (Li et al., 2020). Despite several limitations, different attempts to develop wheat hybrids were made but still, hybrid breeding is not completely established.

During the 1990s, hybrid wheat programs were re-initiated by the CIMMYT-Monsanto joint project. Over the last few decades, global area under hybrid wheat is increasing; in Europe hybrid wheat cultivation impressively increased from 100,000 ha to 560,000 ha during 2017–2018. The success of hybrid wheat production can be witnessed in winter wheat variety ‘Hystar’ produced by the joint collaboration of Germany and Portugal. Moreover, several public and private companies have launched programs for the development hybrid wheat such as CROPCO’s program in the United Kingdom. Syngenta claims for the release first wheat hybrid in India in near future. In China, more than 50 wheat hybrids having 10–20% increased yield potential have been developed; out of these, seven hybrids (Yunza 6, Yunza 3, Jingmai 6, Mianyang 32, Jingmai 7, Yunza 5, Mianzamai 168) were approved. In another study, 3000 wheat lines were evaluated for hybrid wheat production which highlighted the importance of hybrid wheat production targeted at multiple traits (Baenziger et al., 2019). The recent studies suggest that recurrent genomic selection may help in achieving long-term selection gain of hybrid breeding (Rembe et al., 2019). By reducing the cost of hybrid seeds production, a better understanding of heterosis, developing heterotic groups, and incorporating novel technologies for fertility restoration, wheat hybrids can be developed in the future.

## Use of DNA markers in wheat breeding

Research on the development of DNA markers started in the early 1980s (Figure 4). In 1984, the restriction fragment length polymorphism (RFLP) assay was deployed for developing linkage and physical maps and studying the extent of genetic diversity among the wheat genotypes (Chao et al., 1989; Lagudah et al., 1991). Subsequently, PCR-based fingerprinting assays including random amplified polymorphic DNA (RAPD), sequence characterized amplified regions (SCAR), and simple sequence repeats (SSRs) were used for the amplification of desired DNA fragments. These DNA markers were associated with several traits including disease resistance, kernel traits, lodging, vernalization response, etc. (Galiba et al., 1995; Keller et al., 1999a; Keller et al., 1999b; Shahid et al., 2002; Campbell et al., 1999; Rahman et al., 2004; Marza et al., 2006). Consequently, several quantitative trait loci (QTLs) associated with heat stress and drought stress were identified which were located on different chromosomes (Malik and Malik, 2015; Sarkar et al., 2021). The use of DNA markers in marker-assisted breeding paved the way for the identification and selection of desirable genotypes/cultivars. *Vrn*, *Ppd,* and *Rht* were frequently used in marker-assisted selection to develop wheat cultivars/lines. By using RAPD and SCAR molecular markers, *Dn 2* genes linked to Russian aphid resistance were identified which were used in developing new resistant wheat cultivars. The SSRs for *Fusarium* head blight resistance (FHB), pre-harvest sprouting tolerance, and mildew resistance were identified and used in marker-assisted selection in wheat (Del Blanco et al., 2003; Kottearachchi et al., 2006; Tucker et al., 2006). Similarly, SSR markers were also used in marker-assisted backcross breeding (MABB) for introducing traits such as grain protein content and drought tolerance in wheat (Davies et al., 2006; Rai et al., 2018).

Single nucleotide polymorphism (SNPs) is an efficient marker system (Gupta et al., 1999) that is found in abundance throughout the genome. Recently, technological advancements have resulted in the identification of important SNPs linked to traits such as tiller number, spikelets per spike, plant height, spike length, protein content, and grain yield per spike (Marcotuli et al., 2017; Chai et al., 2018; Wang et al., 2018).

## Development and utilization of SNP chips in wheat breeding

The SNPs-based platforms, containing informative SNPs are attractive and powerful tools used for studying the genetic diversity in wheat. The diversity array technology (DArT) was among the initial array-based technologies that generated hundreds of anonymous markers in wheat (Allen et al., 2011). A 9K SNP chip was developed for genotyping 2994 wheat lines. The generated information was used in genome-wide association studies (Cavanagh et al., 2013). The development of Illumina 90K gene-associated SNPs array designed to identify polymorphism in wheat, and KASP assays accelerated the use of SNP markers in wheat breeding (Allen et al., 2011; Wang S. et al., 2014). For detecting polymorphism in primary, secondary, and tertiary pools, an 820 K Affymetrix Axiom SNP array was developed (Winfield et al., 2016). The Wheat Breeders’ 35K Axiom array was later derived from the Wheat 820K SNP array (Allen et al., 2017). Another genome-specific and widely used 660K SNP array was fabricated for studying the polymorphism among the wheat genotypes (Sun et al., 2020). Moreover, some other SNP chips including 15K SNP (Boeven et al., 2016; Qaseem et al., 2019), and 55K SNP developed from 660K has been widely used for GWAS in wheat (Ye et al., 2019; Jin et al., 2020).

## Whole genome sequencing of wheat

The huge size of the wheat genome (∼17.6 Gb) contains approximately 90% repetitive DNA which was the main hurdle in sequencing the whole genome (Li et al., 2004; Wanjugi et al., 2009). The International Wheat Genome Sequencing Consortium (IWGSC) was initiated to sequence the complex genome of wheat (Gill et al., 2004). Finally, in 2012, the first whole genome sequencing information of a Chinese Spring wheat variety “CS42” was released by deploying the short gun whole genome sequencing technique (Brenchley et al., 2012). Afterward, one chromosome at a time was sequenced and the genome assembly of 10.2 billion bases was constructed (IWGSC: The International Wheat Genome Sequencing Consortium, 2014)**.** In the third attempt, 12.7 billion bases were assembled (Clavijo et al., 2017). Eventually, in the year, 2017–2018, the wheat genome of Chinese spring wheat was sequenced and released as the first reference wheat genome. With the rapid advancements in sequencing and assembly tools, many wheat cultivars were resequenced including the Chinese wheat-rye 1RS.1BL translocation cultivar “Aikang 58” (Ru et al., 2020), French bread wheat cultivar “Renan” (Aury et al., 2022), transformation-amenable common wheat cultivar “Fielder” (Sato et al., 2021), etc.

## Genome editing as an emerging technique

Genome editing can specifically modify the genome by inducing insertions, deletions, substitutions, and or targeted mutations (Zhang J. et al., 2018). Earlier genome editing tools such as zinc finger nucleases (ZNFs) and transcription activator-like effector nucleases (TALENs) have been replaced with Clustered Regularly Interspaced Short Palindromic Repeats and associated protein 9 (CRISPR Cas/9). CRISPR/Cas9, a highly precise genome editing tool is used to induce specific double-stranded breaks in the genome (Azhar et al., 2021). This assay is relatively more economical and user-friendly than other editing assays including ZNFs and TALENs (Razzaq et al., 2021). Previously, CRISPR/Cas9 and transcription activator-like effector nucleases (TALEN) were used to knock out Mildew Locus O (*Mlo*) gene for enhancing resistance to powdery mildew (Wang C. et al., 2014).

Due to allohexaploid nature of wheat, it is very difficult to target three or more genes simultaneously. After successful development of stable plants in wheat, α-gliadin genes were targeted to decrease the gluten content (Sánchez-León et al., 2018). Similarly, *TaGW2* homeologs were knocked out to develop mutant lines containing high protein content and grain weight (Zhang Y. et al., 2018). Furthermore, *EDR1* homeologs and *TaEDR1* lines showed resistance to powdery mildew (Zhang et al., 2017). To understand meiotic crossover, *TaZIP4-B2* was targeted (Rey et al., 2018). Likewise, through Agrobacterium-mediated CRISPR/Cas9, each of the *Qsd1* homeo alleles was targeted to suppress pre-harvest sprouting (Abe et al., 2019). Haploid wheat was developed by editing matrilineal (*TaMTL*) that triggers haploid production and centromere-specific histone H3 (*CENH3I*) gene that plays a vital role in the segregation of chromosomes during cell division (Liu et al., 2020b).

## Genomic selection

Genomic selection (GS) helps to select superior genotypes by integrating genotypic and phenotypic data of a training population (TP) to predict breeding values (GEBVs) of a breeding population (BP) (Meuwissen et al., 2001). On the basis of these GEBVs, better performing plants can be selected for use as parent genotypes in the next breeding programs. The similarity index of the molecular marker profile of these individuals with TP allows us to predict the best performing plants. Over time, GS has been efficiently used in wheat breeding due to its high accuracy. In the wheat breeding program, genotype-by-sequencing (GBS) was used to detect polymorphisms followed by the estimation of prediction accuracy (Poland et al., 2012). Relatively, low to moderate prediction accuracy (0.28 and 0.42) was recorded that was higher than the established marker platforms. Another study showed that *Fusarium head blight* resistance showed moderate to high prediction accuracies (0.67–0.82) making it a promising approach for improving resistance to *Fusarium* (Arruda et al., 2015). In GS, the prediction accuracy for grain quality ranged from 0.27 to 0.81, thus showed its potential for deploying in a wheat breeding program (Sandhu et al., 2021).

## Speed breeding—A way to accelerate breeding cycles

Conventional wheat breeding systems take several years to release a stable variety which hinders the breeding progress. However, speed breeding made it possible to harvest up to 4–6 generations of spring/winter wheat per year (Watson et al., 2018). The photoperiod of wheat has been extended to accelerate the developmental stages of plants. Plants grown under controlled environment chambers (speed breeding) reach the anthesis and heading stage almost half the time earlier than the plants grown under natural field conditions. The plant grown under light supplemented glasshouses have been evaluated on the basis of their germination rate.

Speed breeding is instrumental in advancing 5–6 generations per year. Thus, speed breeding can help in lessening the time required for each breeding cycle (Watson et al., 2018). Several protocols have been proposed for undertaking phenotypic characterization of wheat plants which can add synergism to speed breeding. For instance, grain dormancy to tolerate sprouting after harvesting (Hickey et al., 2009, 2010), resistance to stripe rust in wheat (Hickey et al., 2011), screening of root traits for improving adaptation to drought stress (Richard et al., 2015), resistance to yellow spot disease (Dinglasan et al., 2016), and leaf rust resistance traits (Riaz et al., 2016) were targeted through speed breeding in wheat. Several other traits including disease related traits, plant height, root traits, and flowering time have also been screened through speed breeding in wheat (Alahmad et al., 2018; Ghosh et al., 2018). Genomic selection together with speed breeding was used to increase genetic gain (Watson et al., 2019). Recent advancements in high-throughput phenotyping further reduced the obstacles in the progress of plant breeding and genetics. Hence, speed breeding coupled with high-throughput phenotyping can help in discovery of novel and desirable traits in a more sustainable way (Al-Tamimi et al., 2016). For instance, selection of some root and seedling traits through speed breeding helped in rapid selection of mature plants with improved root architecture (Richard et al., 2015).

## High throughput phenotyping

High throughput phenotyping assays have been used to monitor and measure several traits in a large number of plants, simultaneously. These techniques take advantage of the latest automated sensors and imaging tools. Through HTP approaches, non-destructive data can be collected more accurately from trials. The unmanned aerial vehicle (UAV)-based RGB imagery HTP approach was used to estimate wheat plant height (Volpato et al., 2021). The UAV was used to select wheat genotypes for grain yield in early selection cycles (Hu et al., 2020). In addition, complex traits such as lodging were also assessed using a UAV system which proved that HTP can be used to study complex traits (Singh et al., 2019).

## Development of genetically modified wheat

In 1992, the first transgenic wheat conferring tolerance to herbicide was developed (Vasil et al., 1992). The complex nature of the wheat genome together with its acceptability by the public remained a major challenge in extending research on the development of transgenic wheat (Shewry Jones, 2005). Initially, transgenic wheat was developed using the biolistic method. Later on, Agrobacterium-mediated transformation approach was used. In 2004, the first genetically modified wheat round-up ready (MON- 71800) was developed by Monsanto through the introduction of the *CP4 Esps* gene—conferring resistance to glyphosate (herbicide). Several transgenic lines were developed containing *avidin* gene conferring resistance to insect pests (Abouseadaa et al., 2015). Moreover, transgenic wheat lines containing *DREB1A, HDG11, WRKY2, TaSHN1, NAC,* and *bZIP2* were produced which had a high tolerance to drought and increased yield potential (Pellegrineschi et al., 2004; Xue et al., 2011; Li et al., 2016; Bi et al., 2018; Gao et al., 2018; Luang et al., 2018) Similarly, heat resistant transgenic wheat was produced which were overexpressing *TaHsfA6f, TaFER-5B, TaHsfC2a* genes (Xue et al., 2015; Zang et al., 2017; Hu et al., 2018). Some other researchers produced wheat lines conferring resistance to viruses, showing high nutritional quality and improved yield (Sivamani et al., 2000; Xue et al., 2004; Tamás et al., 2009). In 2020, Argentina was the first country to approve drought-resistant GM wheat Bioceres HB4 (Sheridan, 2021). Recently, Brazil also approved HB4 developed by Argentina for consumption as flour.

## Contribution of wheat towards global food security in changing climates

Wheat is consumed by 2.5 billion people as a staple crop and it contributes to global food security by providing 20% calories and proteins. Escalating temperature can severely affect the average wheat yield across the globe. It was projected that a 1°C rise in temperature can suppress yield by 10%. In the coming years, the impact of changing environmental conditions and wheat production to feed extra 3 billion people will be the major challenges for wheat breeders (Curtis and Halford, 2014). The annual yield should be increased to 1.6% to meet the increasing demand of the human population under the scenario of changing environments (Ray et al., 2013; Wheeler and Von Braun, 2013; FAO, 2017). Another major menace is the limited or non-availability of irrigation water which is required for maintaining normal wheat growth. It has been reported that drought alone can reduce yield by up to 86%, and the condition can be worsened under the changing environmental conditions (Shafeeq and Zafar, 2006; Joshi et al., 2007; Prasad et al., 2011). Development of resilient wheat varieties that can demonstrate high yield potential is required for sustaining wheat production worldwide. Also, the changing climate can foster the evolution of new strains of pathogens and diseases. For example, new races/strains of rust disease can overcome the available resistance in cultivated wheat varieties. Rust diseases can cause 15%–20% wheat yield losses worldwide (Figueroa et al., 2018). The incorporation of rust-resistant genes against evolving pathogens into high-yielding wheat varieties is very important for sustainable wheat production. Thus, new resilient wheat varieties under the changing environmental conditions can ensure global food security.

### Increasing grain yield to ensure food security

Grain yield (Y)-a complex quantitative trait-is affected significantly by biotic and abiotic stresses. Grain yield is dependent on biomass (B**)** and grain harvest index (HI) (Yield = B × HI). During the green revolution, HI was improved by about 60% by the incorporation of height-related genes in old wheat varieties. Yield can be improved by increasing photosynthetic area or capacity (Parry et al., 2011). Canopy architecture, large-spike, and spike fertility can also contribute to high yield (Gaju et al., 2009; Murchie et al., 2009; Reynolds et al., 2009). For pyramiding all these traits in one cultivar, DNA markers can be used for monitoring the introgression of these traits. Likewise, wheat hybrid breeding can also enhance wheat production in the future.

### Eradication of malnutrition by quality improvement

Micronutrient deficiency is also a major challenge, almost three billion people are affected by these deficiencies globally (Welch and Graham, 2004). Children and females in developing countries are more prone to Zinc (17%) and Iron (33%) deficiencies worldwide (Wessells and Brown, 2012; Kassebaum et al., 2014). To address micronutrient deficiencies, wheat is the best candidate crop as it is consumed by a large population globally. It has been reported that Zn and Fe concentration is relatively high in closely related wild wheat species (Çakmak et al., 2004). Provitamin content has been increased by expressing bacterial *CrtB* and *CrtI* gene through transgenic methods. Similarly, protein content has been enhanced by expressing *Amaranthus albumin* gene and Fe content by the soybean *ferritin* gene in wheat (Cong et al., 2009; Wang C. et al., 2014). However, varieties expressing high-quality traits have relatively low yield potential. For wider acceptability, support price (premium) for such varieties should be announced by the regulators for encouraging their cultivation. Alternatively, some transgenic wheat lines expressing high Zn and Fe contents should be allowed for cultivation in restricted parts of wheat-growing countries that can be mixed in flour of non-transgenic wheat varieties.

### Enhancing resilience to stresses

Development of varieties having high yield potential and resilience to stresses is the need of the hour (Juliana et al., 2019). Wild relative of wheat such as *Aegilops tauschii* (DD) is a good source for climate resilience because it can easily be crossed with durum (AABB) or bread wheat (AABBDD) to generate synthetic wheat (Elbashir et al., 2017). Hybrid wheat is another promising approach as it has higher yield stability and tolerance to stresses. Hybrids in wheat have been produced with resistance to *Fusarium head blight*, frost resistant, leaf rust resistance and, *Septoria tritici* blotch resistance showing hybrid wheat potential with context to climate change (Longin et al., 2013; Miedaner et al., 2017).

## Conclusion

Wheat is one of the ancient crops and it is the crop of the future. Since its domestication, breeders and farmers have modified wheat continuously through selections and by incorporating different genes for short plant height, and biotic and abiotic stresses. Despite the extensive research, still there is a gap between the total wheat production and consumption, particularly in developing countries. The current rate of genetic gain is alarming which would not help in meeting the food demand of the growing human population in 2050. Changes in climatic conditions may further worsen the situation by inviting new pests and diseases, reducing yield due to terminal heat, and altered rainfall patterns may reduce the cultivated area of wheat in several countries. Under such circumstances, genes conferring resilience to rust diseases, terminal heat, drought, and salinity are required to be introduced into wheat cultivars. For increasing yield potential by 30%, it is extremely important to find new genetic solutions for tackling the issue of male sterility and restoration in hybrid wheat. For example, the adoption of new technologies including high throughput phenotyping, gene editing, speed breeding, molecular breeding, and selection strategies can accelerate the magnitude of genetic gains of the newly developed varieties. Thus, wheat production can be sustained beyond 2050.
